# Agents and Patients in Physical Settings: Linguistic Cues Affect the Assignment of Causality in German and Tongan

**DOI:** 10.3389/fpsyg.2017.01093

**Published:** 2017-07-07

**Authors:** Andrea Bender, Sieghard Beller

**Affiliations:** Department of Psychosocial Science, Faculty of Psychology, University of BergenBergen, Norway

**Keywords:** causal cognition, causal attribution, agency, language, ergative case, implicit verb causality, culture, Tongan and German

## Abstract

Linguistic cues may be considered a potent tool for focusing attention on causes or effects. In this paper, we explore how different cues affect causal assignments in German and Tongan. From a larger screening study, two parts are reported here: Part 1 dealt with syntactic variations, including word order (agent vs. patient in first/subject position) and case marking (e.g., as ergative vs. non-ergative in Tongan) depending on verb type (transitive vs. intransitive). For two physical settings (wood floating on water and a man breaking a glass), participants assigned causality to the two entities involved. In the floating setting, speakers of the two languages were sensitive to syntactic variations, but differed in the entity regarded as causative. In the breaking setting, the human agent was uniformly regarded as causative. Part 2 dealt with implicit verb causality. Participants assigned causality to subject or object of 16 verbs presented in minimal social scenarios. In German, all verbs showed a subject (agent) focus; in Tongan, the focus depended on the verb; and for nine verbs, the focus differed across languages. In conclusion, we discuss the question of domain-specificity of causal cognition, the role of the ergative as causal marker, and more general differences between languages.

## Introduction

Physical situations look the same all over the world. They follow invariable laws of nature and appear to be open to direct inspection, irrespective of the culture or language of a potential observer. But do people *represent* them and *reason* about them in the same way everywhere in the world? While causal cognition has been subject to a great deal of exploration over the last two millennia, specifically in philosophy and psychology (for an overview, see Waldmann and Hagmayer, 2013), the potential for cultural and linguistic diversity has attracted far less interest (Bender et al., 2017, for exceptions, see the contributions to Beller et al., 2014).

Previous research points to a small number of factors that—even within cultural and/or linguistic groups—may affect causal cognition, not only in the social domain but also in the physical domain: biases in assigning causality, specific causal concepts, and linguistic cues. The first group of these factors generally skews the assignment of causality a priori: The causal asymmetry bias (White, 2006), for instance, leads people to assign the roles of cause and effect to entities even in symmetric interactions, and to overestimate the contribution of the assumed cause entity to the overall interaction. Being a domain-general feature of causal cognition, this bias affects most of what people perceive, believe, and linguistically express with regard to causal relations, and even restrains research questions and methods (White, 2006, 2007; Bender and Beller, 2011). The second group of factors is restricted to specific settings, but is still pervasive, namely the causal theories of folk science (Keil, 2003; Lawson, 2006), including such popular misconceptions as the impetus concept in motion (McCloskey, 1983; Hubbard and Favretto, 2003), or the valve model of how a thermostat functions (Kempton, 1986). They are responsible for content effects detected, for example, with different content versions of otherwise identical tasks (Cummins, 1995; Beller and Spada, 2003; Beller and Kuhnmünch, 2007; Klauer et al., 2010; and see Le Guen et al., 2015). The third—and for the purpose of this paper most relevant—group of factors involves variations in linguistic cues that may be employed distinctively to shift attention to cause or effect. Assignment as prime cause may be affected, for instance, by a shift in what is focused on as figure and what as ground (Kuhnmünch and Beller, 2005; Beller and Bender, 2015). Conveying this focus involves language, at least as a medium, and its effectiveness thus testifies, rather non-controversially, to an impact of language on causal representations.

Besides such distinct cues from within a given language, however, diverging properties of different languages might also play a role in shifting attention in a specific manner. Cross-linguistic studies reveal that languages differ in how they encode information about causal relations and events (e.g., Ikegami, 1991; Wolff et al., 2009; Bohnemeyer et al., 2010; Fausey and Boroditsky, 2011), but the question of whether such language-specific grammatical features and phrasing preferences entail cross-linguistic differences in cognitive representations of causality has barely been investigated. We therefore attempted to address this question by exploring the influence of two types of linguistic cues on the assignment of causality in two non-related languages: German, which belongs to the Indo-European language family and serves as mother tongue to a 100 million people, and Tongan, which belongs to the Austronesian language family and is spoken by the approximately 100,000 inhabitants of the Polynesian Kingdom of Tonga in the Southwest Pacific.

Mainstream research is still guided by the wide-spread assumption that causal cognition tends to be universal, specifically in the physical domain. Given the resultant shortage in empirical evidence, we conducted a screening study with the main purpose of exploring potential cultural and linguistic impacts on causal cognition in the physical domain (Bender and Beller, 2011). The data presented here were collected as part of this screening, and address the question on whether grammatical features and phrasing preferences may affect causal representations. More specifically, we investigated within-language effects of content domain (physical setting), verbs and verb type (transitive vs. intransitive), and word order, but also between-language effects of different grammatical structures (nominative-accusative vs. absolutive-ergative). In the following, we first provide a theoretical background on the linguistic coding of information about causal relations and events, before we motivate and present the current study.

## Linguistic coding of causality

Linguistic descriptions have long been known to affect how people represent a described event—even when they eye-witnessed it themselves (e.g., Loftus and Palmer, 1974, and see Fausey and Boroditsky, 2011). This is the very reason why particular care has to be taken with regard to how inquiries are phrased, for instance in court.

In principle, a causal relationship can be understood as an event, caused by one entity (the *causer* or *agent*) and affecting another entity (the *effect* or *patient*). Typically, if not necessarily, agents are conceived of as being animate, sentient, moving, instigating and controlling the respective action or causing the respective event (Langacker, 1987). However, according to Dowty (1991, p. 572), *agency* is a prototype rather than an either-or concept, clustered around a set of properties that include (i) volitional involvement in the event or state, (ii) sentience and/or perception, (iii) causation of an event or change of state in another participant, (iv) movement relative to the position of another participant, and (v) existence independently of the event named by the verb. While human beings combine these properties in a paradigmatic manner, the same set of properties can also be recruited for assigning agent and patient roles in non-human, entirely physical settings such as the launching of an object by another object (Mayrhofer and Waldmann, 2014).

When translating a causal relationship into language, the notion of causality can be linguistically encoded in numerous ways and across different elements of a clause, for instance in syntactic categories such as subject, in verb semantics, in morphology, in resultative constructions, or in animacy distinctions as coded in noun phrases (for examples, see Duranti and Ochs, 1990; Ikegami, 1991; Wierzbicka, 2002; Wolff and Song, 2003; Wolff, 2007; Bohnemeyer et al., 2010). Here, we are particularly interested in two types of linguistic variations: cues derived from the syntactic structuring around the verb, and causal information implicit in verb semantics.

### Syntactic structuring

**Figure 1:**
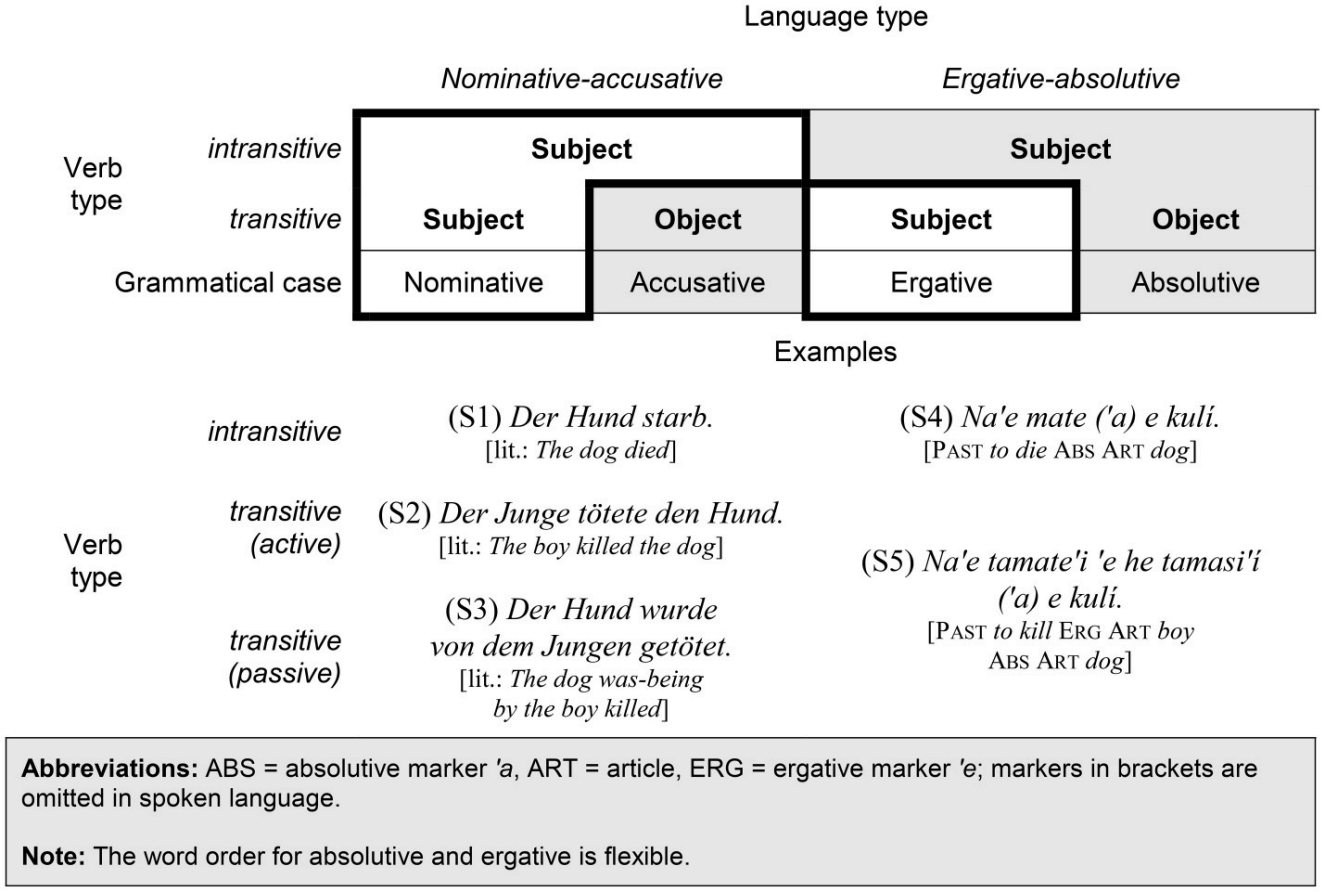
Categorization of subject and object in nominative-accusative languages and ergative-absolutive languages, with examples from German and Tongan (adapted from Bender and Beller, 2011).

The structure of a sentence is determined, at least to a considerable extent, by its core component: the verb. For our purpose, two types of verbs will be contrasted: *transitive verbs* like “kill,” which entail subject and object, and *intransitive verbs* like “die,” which entail a subject only. Mostly, although not necessarily, the semantic roles of agent and patient figure syntactically as *subject* and *object*, at least in active sentences. Yet, how these syntactic roles are categorized differs across languages (cf. Figure 1).

In *nominative-accusative languages* like German or English, subjects of intransitive and transitive verbs are treated uniformly, and are distinguished from objects of transitive verbs by their case: nominative for the subject (i.e., *der Hund* [“the_NOM_ dog”] in S1 and *der Junge* [“the_NOM_ boy”] in S2) and accusative for the direct object (i.e., *den Hund* [“the_ACC_ dog”] in S2). Although it is typically the agent who figures as the subject, this need not be the case: The patient can also hold the subject position indicated by the nominative case, for instance if the verb is transformed into the passive voice (as with *der Hund* [“the_NOM_ dog”] in S3).

*Ergative-absolutive languages* like Tongan, on the other hand, distinguish subjects of intransitive verbs from subjects of transitive verbs (Plank, 1979; Dixon, 1994; Manning, 1996). The former have the same grammatical case (i.e., absolutive) as objects of transitive verbs, whereas the latter are put in the ergative case. Therefore, '*a e kul*í (“abs the dog”) has the same grammatical form both in S4 and S5, whereas '*e he tamasi*'í (“erg the boy”) in S5 is highlighted with the ergative marker '*e*.

From a propositional point of view, S2, S3, and the respective ergative sentence S5 are equivalent. Yet, S3 is marked by the passive voice (in contrast to the unmarked active voice in S2), while its complement in an ergative-absolutive language (S5) is marked by the ergative case of the transitive subject (in contrast to the unmarked absolutive case of intransitive subjects and transitive objects). In other words, nominative-accusative languages categorize according to *focus*, whereas ergative-absolutive languages categorize according to the *entities undergoing a change of state*.

Only a small number of studies have so far examined the cognitive implications of these syntactic variations. For instance, Goldin-Meadow (2003) reports that, irrespective of their mothers' language, deaf children are more likely to spontaneously produce gestures for intransitive agents and for patients than for transitive agents, thus exhibiting an ergative pattern. For speakers of Samoan, an ergative language closely related to Tongan, a similar focus on intransitive agents and patients was observed, at least in socio-political discourse (Duranti, 1994).

**Figure 2:**
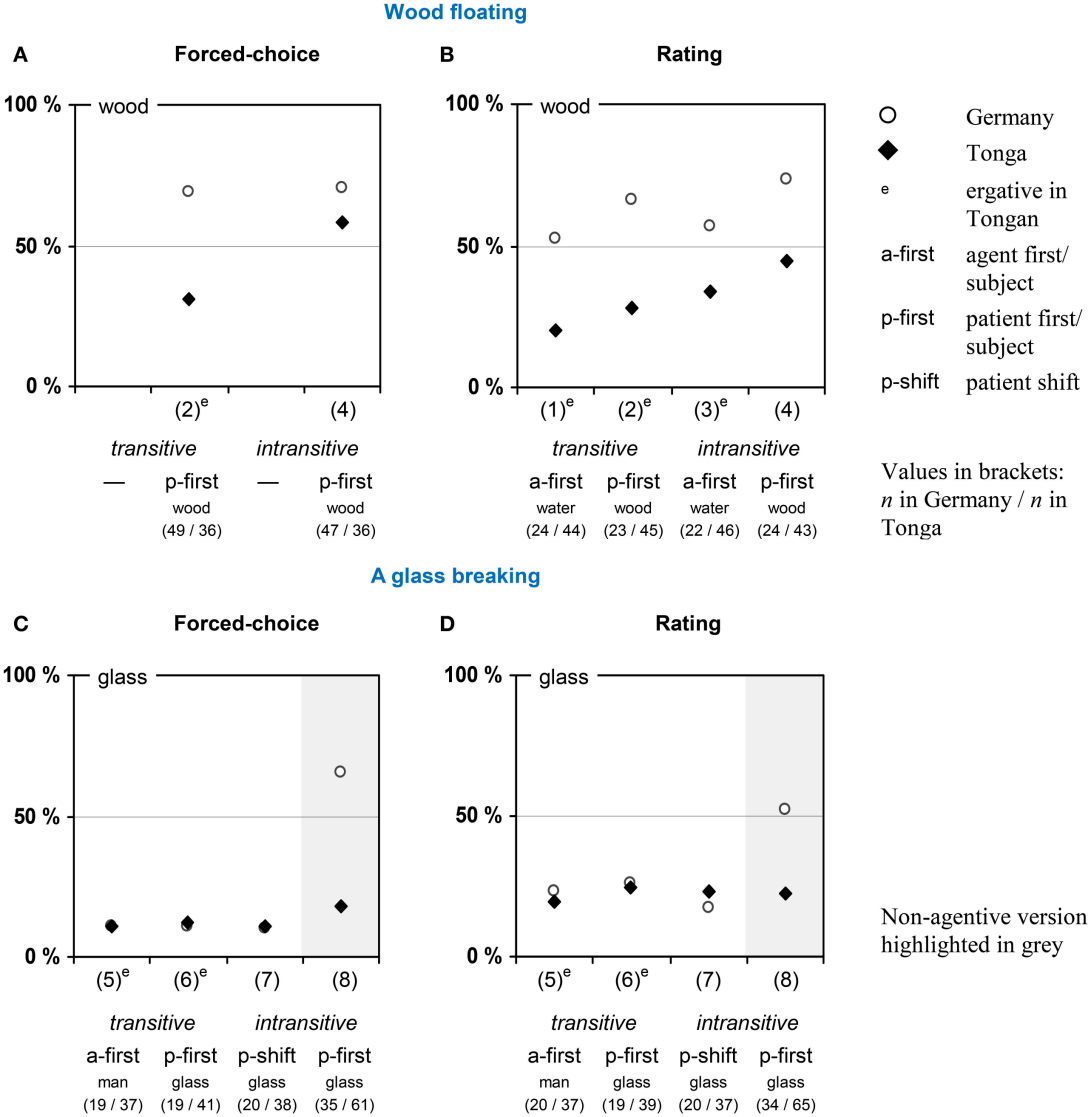
Assignment of causality in Part 1 on syntactic variations. The data presented in **(A)** are taken from Beller et al. (2009b, Table 1) and are included for reasons of comparison.

Now, if speakers of ergative languages are, by default, largely content with providing and receiving information about the action and the entity affected, then introducing a transitive agent and marking him or her with the ergative case by way of exception might serve as a particularly potent tool for agency assignment. A cross-linguistic experiment (Beller et al., 2009b) explored this hypothesis by testing whether the ergative does indeed shift agency assignment in a symmetric physical setting that does not involve a “proper” semantic agent (i.e., no animate entity). The experiment contrasted the intransitive phrasing “wood floats on water” and the transitive passive phrasing “wood is carried by water” in German, with respective changes in case marking as absolutive vs. ergative in Tongan. The change from intransitive to transitive increased the assessment of water as causative (the agent marked by the ergative) in Tongan, but not in German, where wood remained in the focus (Figure 2A), suggesting that the ergative marking in Tongan may indeed provide a stronger tool for indicating agency than its counterpart in German.

However, due to the different structures of the languages, the two sentences were not entirely comparable. More specifically, in order to keep word order constant across languages, the transitive construction in German had to be phrased with the (marked) passive voice. Adding an (unmarked) active phrasing that allows us to disentangle their relative effects would thus be required to justify any strong conclusion.

Two further reservations may be raised with regard to this previous study: First, it used a forced-choice response format, which may have distorted responses in an unintended way. Second, and more importantly, the floating setting is purely physical and symmetric; assigning the thematic role of the agent to either entity involved may therefore be problematic. With the data reported in this article, we aim to remedy these reservations by comparing responses assessed using the forced-choice format with responses assessed using an analog rating scale, and by comparing a purely physical setting with a setting that involves a human agent.

This latter setting also allows us to contrast agentive phrasings with a non-agentive phrasing (Hare et al., 2009; Fausey and Boroditsky, 2011). *Agentive* phrasings are typically transitive and indicate an agent in the subject position, as in “He broke the glass,” whereas *non-agentive* phrasings are intransitive, with the entity affected (patient) in the subject position, as in “The glass broke.” In line with related work (e.g., Ikegami, 1991), Fausey and colleagues demonstrated that speakers of different languages differ with regard to their preferences for agentive vs. non-agentive phrasings (Fausey et al., 2010; Fausey and Boroditsky, 2011), and that these preferences also affect their causal assignments: The more an event is described in an agentive way, the more likely the (personal) agent will be blamed (Fausey and Boroditsky, 2010).

Importantly, though, by virtue of its subject position, people might be willing to consider the affected entity to be the “agent” of the intransitive verb, and might thus assign more causality to it. If this tendency is further emphasized by case marking, it should be stronger in nominative-accusative languages, which shift “the glass” from (accusative) object to (nominative) subject, than in ergative-absolutive languages, in which “the glass” remains in the absolutive case.

### Implicit verb causality

Assigning causal roles to the entities involved in a specific relation is likely the most relevant objective in identifying its causal structure. While this seems to prioritize the entities as the main source of information relevant for role assignment, the relation itself and its linguistic representation through a specific verb plays an equally important, albeit perhaps more subtle, role in this process—and again, Loftus and Palmer's (1974) classical study on motion events may serve as a striking case in point: Participants estimated the speed of two cars involved in an accident differently, depending on the verb used in the target question (e.g., “contact,” “hit,” or “smash”).

Since Abelson and Kanouse (1966) reported the phenomenon later called “implicit verb causality,” it has been demonstrated repeatedly that different verbs used to describe abstract interpersonal events may give rise to different causal assignments (for overviews, see e.g., Rudolph and Försterling, 1997; Ferstl et al., 2011). The verb “cheat,” for instance, is conceived of as being primarily in the responsibility of the agent, whereas “congratulate” rather suggests that the congratulation was evoked by the person congratulated (i.e., the patient) and by something he or she has accomplished.

This difference in focus on subject vs. object can be used to establish taxonomies of interpersonal verbs, first in *state* and *action verbs*, and then further into subtypes, depending on the underlying theory. For instance, in Au's (1986) terminology, “cheat,” with its subject focus, would be regarded as an *action-agent verb*, whereas “congratulate” would be considered an *action-patient verb*, due to its object focus (for alternative taxonomies, see also Brown and Fish, 1983; Semin and Fiedler, 1991; Crinean and Garnham, 2006). Although this field of research has focused on implicit causality, recent studies have proven that verbs also differ with regard to whether they are more likely to trigger causal or consequential inferences (Majid et al., 2007; Pickering and Majid, 2007).

Given our primary interest in physical causality, our intention with the study reported below was not to systematically explore verb causality in German and Tongan (for respective studies on German see, e.g., Fiedler and Semin, 1988; Rudolph and Försterling, 1997), but to assess the potential for interferences with causal assignments. The main question was whether the verb itself, even in the absence of any concrete context or information on the entities involved, would already shift participants' assignments in a language-specific manner. Due to our focus on the physical domain, we selected verbs that can refer to physical settings, but had no particular expectation regarding potential cross-linguistic differences. In contrast, for a small group of verbs referring to social contexts, we did have reasons to expect such differences. Tongan culture places a strong emphasis on cooperation and sharing with others (*fetokoni*'*aki*), and granting other people their requests—within certain limits—is regarded as a core value (Morton, 1996; Evans, 2001; Bender, 2007). The clearer a request is articulated, the more compelling is the obligation (Beller et al., 2009a). Given this cultural evaluation, respective transaction verbs such as “giving,” “offering,” or “helping” may thus have a stronger object focus in Tongan than in German.

## The study

The data presented in the current article were collected as part of a larger screening, which aimed at exploring the potential influences of culture on causal cognition and consisted of several sections. One section of the screening asked participants to assign causality in a range of purely physical, symmetric settings, varying content and focus (reported in Bender and Beller, 2011). Another section was concerned with potential linguistic effects on causal assignments, on both the syntactic and the semantic level (reported in this article), and a final section was concerned with causality as cognitive determinant for emotions (not considered here).

### Methods

The two linguistic objectives of the screening, which are the subject of the current article, will be referred to as Part 1 (syntactic variations) and Part 2 (implicit verb causality), respectively.

The tasks in Part 1 aimed at assessing how syntactic variations affect the assignment of causality. The prime goal was to replicate a main finding of a previous study (Beller et al., 2009b)—namely that ergative case marking in Tongan shifts agency assignments, and does so more strongly than the passive transformation in German—and to broaden the empirical basis by additional variations and different response formats. More specifically, we hypothesized that re-phrasing an agentive (transitive) sentence as non-agentive (intransitive) does shift causal assignments toward the non-agentive subject. This effect should occur in both languages, but should be more pronounced in nominative-accusative languages than in ergative-absolutive languages due to the concurrent shift in case marking in the former but not the latter. A second goal was to explore the effect of including a full-fledged semantic (human) agent. We hypothesized that if such an agent is present, this agent should strongly attract causal assignments across other syntactic variations.

Part 2 aimed at assessing how verb semantics affect the assignment of causality. Given that implicit verb causality has never before been investigated for a Polynesian language like Tongan, we also intended to probe the potential of cross-linguistic variability in this regard. Specifically, we wanted to explore whether verbs that could be used to describe (symmetric) physical relations exhibit a subject or object focus in the first place, and do so distinctively in different languages. For verbs focusing on social events related to the Tongan obligation to help (*fetokoni*'*aki*) we hypothesized a more pronounced object focus than other verbs in Tongan, and more so than their German counterparts.

#### Materials

##### Part 1: Syntactic variations

In this part, two physical settings were used: Wood floating and a glass breaking. For each setting, four syntactic variants were constructed by crossing *verb type* (transitive vs. intransitive) and *word order* (agent vs. patient in first/subject position; see Table 1).

**Table 1:** Syntactic variants used in Part 1 on syntactic variations (with English translations).

	**Wood floating**	
	**Word order**	
**Verb type**	**Agent first/subject: Water *(Wasser, vai)***	**Patient first/subject: Wood *(Holz, papa)***
Transitive (carrying)	(1) The fact that water carries wood, … Ge: *Dass Wasser Holz trägt,…* To: '*Oku 'ave 'e he vai 'a e papa,…*^e^	(2) The fact that wood is carried by water, … Ge: *Dass Holz von Wasser getragen wird …* To: '*Oku 'ave 'a e papa 'e he vai,…*^e^
Intransitive (floating)	(3) The fact that water lets wood float, …^a^ Ge: *Dass Wasser Holz schwimmen lässt,…* To: '*Oku tukuange 'e he vai ke tētē 'a e papa,…* ^e^	(4) The fact that wood floats on water, … Ge: *Dass Holz auf Wasser schwimmt,…* To: '*Oku tētē 'a e papa 'i he vai,…*
	**A glass breaking**	
	**Word order**	
**Verb type**	**Agent first/subject: Man** ***(Mann, tangata)***	**Patient first/subject: Glass** ***(Glas, sio'ata)***
Transitive (breaking_1_)	(5) The fact that the man breaks the glass, … Ge: *Dass der Mann das Glas zerbricht,…* To: '*Oku fahi 'e he tangata 'a e sio'ata,…*^e^	(6) The fact that the glass is broken by the man, … Ge: *Dass das Glas von dem Mann zerbrochen wird,…* To: '*Oku fahi 'a e sio'ata 'e he tangata,…*^e^
Intransitive (breaking_2_); *patient shift*		(7) ^*^The fact that the glass breaks to the man, …^b^ Ge: *Dass das Glas dem Mann zerbricht,…* To: *Ko e hoko 'a e mafahi 'a e sio'ata ki he tangata*, …
Intransitive (breaking_2_); *non-agentive*		(8) The fact that the glass breaks, … Ge: *Dass das Glas zerbricht,…* To: '*Oku mafahi 'a e sio'ata,…*

*Ge: German; To: Tongan*.

a *Variant (3) somewhat strains the notion of an intransitive sentence with water as subject: Water is subject only with regard to “let,” while wood still remains the subject for the (intransitive) “floating.” Yet, this ‘split agency’ was the very reason for including this variant*.

b *Although this phrasing would not be used in English, it is canonical in German and feasible in Tongan*.

e *Ergative construction*.

For the floating setting, a purely physical setting without a human agent, the following variants were used:

Transitive, agent first/subject: “Water carries wood.”                     (1)
Transitive, patient first/subject: “Wood is carried by water.”                     (2)
Intransitive, agent first/subject (split agency): “Water lets wood float.”                     (3)
Intransitive, patient first/subject: “Wood floats on water.”                     (4)

As not all combinations of verb type and word order could be filled with a one-verb phrasing, we decided to choose a construction with “let” that splits agency for variant (3): *Wood* serves as the subject for the (intransitive) “floating” and thus, in a loose sense, as the agent in this specific activity, while part of the agency is shifted to the *water*, which is subject with regard to “let.” Hence, we classified this sentence as intransitive with agent (water) in the first/subject position.

Three of the Tongan sentences required an ergative construction: The split agency phrasing (3) and the transitive phrasing (1), both of which emphasize the agent by word order and subject position, but also the transitive phrasing (2) that emphasizes the patient. The reason for this is that the passive transformation used in English and German to implement variant (2) is not possible in Tongan; the closest we can get is a phrasing as in (1), yet with reversed word order (Churchward, 1953, p. 67f.). As a consequence, the shift in word order from (1) to (2) implies a shift in case marking in German, but not in Tongan.

For the breaking setting, the following four variants were used, three of which explicated a human agent:

Transitive, agent first/subject: “The man breaks the glass.”                     (5)
Transitive, patient first/subject: “The glass is broken by the man.”                     (6)
Intransitive, patient first/subject (patient shift): “^*^The glass breaks to the man.”                     (7)
Intransitive, patient first/subject (non-agentive): “The glass breaks.”                     (8)

One slot (intransitive verb with agent in first/subject position) was again impossible to fill. For explorative purposes, we therefore decided to include construction (7) with the patient in first/subject position for which German marks the agent by the *dativus commodi* case and thus reverses the typical causal relation, indicating the man as the entity being affected. The respective sentence was thus classified as intransitive with *patient shift*. From a linguist's point of view, the intransitive verb still does not render “the glass” the agent; speakers of German might nonetheless feel inclined to consider it to be agentive to a certain extent. Note also that variant (8) mentions the patient, but leaves the agent unnamed and is thus *non-agentive*.

Two of the Tongan sentences required an ergative construction (one, again, because the passive used in German is not possible in Tongan): The transitive phrasing (5) in which word order and subject position emphasized the agent, but also the transitive phrasing (6) in which the two factors emphasized the patient.

*Assessment of causal assignments*: All target items were formulated using the following sentence frame, exemplified for variant (1) (the complete list of syntactic variants is presented in Table 1):

“The fact that water carries wood is basically due to …
the water |----------------------------| the wood.”
German:
“Dass Wasser Holz trägt, liegt vor allem …
am Wasser |----------------------------| am Holz.”
Tongan:
“'Oku ave 'e he vai 'a e papa, ko e tupu mei …
he vai |----------------------------| he papa.”

In the floating setting, causal assignments were assessed with an analog rating scale of 10 cm length, which allowed for the allocation of relative causal effectiveness. Each side of the scale was labeled with one of the two entities “the water/the wood.”

The four syntactic variants of the breaking setting were implemented each in two assessment versions: first with a forced-choice format that simply required participants to decide which of the two entities in question is the main cause for the overall event (e.g., “…□ the man; □ the glass.”), and second with an analog rating scale of 10 cm length in order to assess the relative causal effectiveness of the two entities (as in the example above). For all variants involving a person—phrasings (5), (6), and (7)—the entities were “the man/the glass” (German: *am Mann/am Glass*; Tongan: *he tangata/he sio'ata*), whereas for the non-agentive variant (8), the entities were formulated either by referring to the glass vs. an unknown person (German: *am Glas/an jemand Unbekanntem*; Tongan: *he sio'ata/he tokotaha ta'e'iloa*) or by referring to the glass vs. an unknown non-personal factor (German: *an etwas Unbekanntem*; Tongan: *he me'a ta'e'iloa*).

##### Part 2: Implicit verb causality

This part aimed at assessing how the verb itself—in the absence of any context information—affects causal assignments. It comprised 16 verbs, which are presented in Table 2. Twelve verbs can be used to describe physical relations; they were examined here to assess a baseline of verb semantics for exploring its potential influence on causal assignments. The remaining four verbs refer to social transactions of giving and helping, which are linked to the core value of *fetokoni'aki* in Tonga and are thus of particular cultural salience.

**Table 2:** List of verbs used in Part 2 on verb semantics (with English translations).

	**English**	**German**	**Tongan**
1	[S] attracts [O].	[S] zieht [O] an.	Tohoaki'i 'e [S] e tokanga 'a [O].^e^
2	[S] interrupts [O].	[S] unterbricht [O].	Fakaheleleu 'a [S] kia [O].
3	[S] resembles [O].	[S] ähnelt [O].	To'onga tatau 'a [S] mo [O].
4	[S] repels [O].	[S] stößt [O] ab.	Fakafepaki 'a [S] kia [O].
5	[S] approaches [O].	[S] nähert sich [O].	Fakaofiofi 'a [S] kia [O].
6	[S] distracts [O].	[S] lenkt [O] ab.	Uesia 'e [S] e tokanga 'a [O].^e^
7	[S] pushes [O] forward.	[S] schiebt [O] nach vorne.	Teke'i 'e [S] 'a [O] ki mu'a.^e^
8	[S] lets [O] swim.	[S] läßt [O] schwimmen.	Tukuange 'e [S] ke kakau 'a [O].^e^
9	[S] carries [O].	[S] trägt [O].	Fua 'e [S] 'a [O].^e^
10	[S] stops [O].	[S] stoppt [O].	Ta'ofi 'e [S] 'a [O].^e^
11	[S] displaces [O].	[S] verdrängt [O].	Fetongi 'e [S] 'a [O].^e^
12	[S] hits [O].	[S] stößt [O] an.	Tā'i 'e [S] 'a [O].^e^
A	[S] gives [O] a book as a present.	[S] schenkt [O] ein Buch.	'Oange 'e [S] 'a e tohi ko e me'a'ofa kia [O].^*e*^
B	[S] gives [O] a picture.	[S] gibt [O] ein Bild.	Foaki 'e [S] 'a e fakatÄtātā'a [O].^e^
C	[S] offers [O] some cake.	[S] bietet [O] Kuchen an.	'Oange 'e [S] 'a e me'i keke 'a [O].^e^
D	[S] helps [O] with the work.	[S] hilft [O] bei der Arbeit.	Tokoni 'a [S] kia [O] ki he ngāue.^e^

*S, subject; O, object*.

e *Ergative construction*.

*Assessment of causal assignments*: All target items were presented as minimal social scenarios of the type “[S_(Subject)_] verb [O_(Object)_]” as shown in the following example:

“Peter carries Anna. This is surely due to
Peter |------------------| Anna.”
German:
“Peter trägt Anna. Das liegt sicher an
Peter |------------------| Anna.”
Tongan:
“Fua 'e Pita 'a 'Ana. 'Oku mahino ko e tupu mei
Pita |------------------| 'Ana.”

The roles [S] and [O] were replaced by proper names that are common in the respective languages. Causal assignments were assessed with an analog rating scale of 5 cm length. The subject [S] was always placed on the left side of the scale and the object [O] on the right side to ensure coherence with the word order in the sentences.

#### Participants

The German sample consisted of 93 students from the University of Freiburg (36 male, 56 female [1 did not indicate his or her gender]; mean age 23.7 years, *SD* = 5.19, *range:* 18–43 years). Compared to the data reported in Bender and Beller (2011), we were able to extend our Tongan sample by 76 to now 179 participants, mostly students from three different high schools (80 male, 93 female [6 did not indicate their gender]; mean age 17.5 years, *SD* = 3.91, *range:* 14–49 years). All participants were native speakers of either German or Tongan, respectively, and none had prior experience with these types of tasks.

Please note that, although the German participants are older than the Tongan ones, the two samples are roughly comparable in terms of education level, as most German participants were shortly after the exams that qualify for university entry, while the Tongan ones were shortly before these exams. Potential implications of the age difference are picked up in the discussion.

#### Procedure and design

Although our university ethics board only deals with medical research, we can confirm that we follow the Frankfurt declaration of ethical conduct for anthropological research, which addresses all stages of the research project from designing to reporting the research.

The study was implemented as a paper-and-pencil questionnaire. The questionnaire always began with general instructions, followed by one task from the breaking setting in forced-choice format. This task was followed by the block of tasks on the content and focus variations reported in Bender and Beller (2011). All subsequent tasks then used the rating format: One task from the floating setting, a second task from the breaking setting, and then the 16 tasks on implicit verb causality. The final part of the questionnaire, which is not considered here, dealt with emotions.

This order of tasks was chosen for three reasons: (a) The two tasks from the breaking setting each participant had to work on were separated from one another maximally in order to minimize (trivial) transfer effects. (b) The task with the forced-choice format always preceded those with rating format, because the former used the more coarse-grained measure. (c) Finally, in the succession of tasks with rating format, the task from the floating setting was always presented before the second task from the breaking setting, because the latter introduced a human agent, and we tried to prevent possible carry-over effects from the setting richer in information to the setting with less information.

The four tasks of the breaking setting with forced-choice format varied between subjects, and the same applied for the four tasks of the floating setting and the four tasks of the breaking setting with rating format. All possible task combinations were implemented, with one constraint: When participants had received an agentive version for the first assessment of the breaking setting, that is, phrasing (5), (6), or (7), they then did not receive the non-agentive version (8) for the second assessment, in order, again, to prevent possible carry-over effects from the information-rich setting.

The order of the response options in the forced-choice format and the orientation of the rating scale in the rating format were balanced across conditions. In the non-agentive version (8), half of the participants received a personal option (“somebody unknown”) and the other half a non-personal option (“something unknown”) as alternative to “the glass.”

The tasks of Part 2 on verb semantics (see Table 2) were always administered after Part 1 had been completed. Participants had to work on all 16 tasks. The physical verbs and the social verbs were presented in blocks, with the block of physical verbs (1–12) always preceding the block of social verbs (A to D). Within each block, different random orders were implemented. In total, four different sequences of tasks were used. Half of the names used were male and the other half were female. Moreover, for each verb, a female name figured as subject and a male name as object for half of the time, and vice versa for the other half. The combination of names and verbs was randomized.

Participants were randomly assigned to the different versions of the questionnaire. They were instructed to respond spontaneously and were given as much time as they needed.

### Results and discussion

The data and findings are presented and discussed in the following order: First, we analyse the data from Part 1 on the effects of the syntactic variations and possible language differences, beginning with the floating setting, followed by the breaking setting. We then turn to Part 2 and compare implicit verb causality across languages and stimuli, before briefly addressing some possible reservations.

#### Causal assignments in the floating setting

Participants' causal assignments were coded by measuring their marks on the rating scale accurate to 0.5 mm ranging from 0 cm (0% wood) when the mark was precisely on the endpoint labeled with “water,” to 10 cm (or 100% wood) when the mark was precisely on the endpoint labeled with “wood.” Accordingly, values above 50% indicate a stronger causal role of the patient (wood) and values below 50% indicate a stronger causal role of the agent (water).

With the floating setting, we aimed at assessing effects of syntactic variations on causality assignments within and across two languages in a purely physical setting without a human agent. The descriptions used either a transitive or an intransitive verb and emphasized either the agent or the patient by word order and subject position, respectively. Across languages, we found an overall preference for the patient (wood) as causative in Germany and for the agent (water) in Tonga as well as significant effects of word order and verb type. Within languages, we found a somewhat stronger preference for the agent if emphasized by word order (in German) or in transitive phrasings (in Tongan) than in the respective complementary conditions (Figure 2B^1^).

An analysis of variance with *verb type* (transitive vs. intransitive), *word order* (agent vs. patient in first/subject position), and *language* (German vs. Tongan) as independent variables and the rating of wood-as-causative as dependent variable (ranging from 0 to 100%) indicated a main effect of language [*F*_(1, 263)_ = 45.74; *p* < 0.001; η_*p*_^2^ = 0.148], of word order [*F*_(1, 263)_ = 7.04; *p* = 0.008; η_*p*_^2^ = 0.026], and of verb type [*F*_(1, 263)_ = 5.55; *p* = 0.019; η_*p*_^2^ = 0.021], without any interactions.

Aggregated across conditions, the German participants preferred the patient/wood as causative for the floating with an average rating of 62.2% (95% CI: 55.0; 69.4), whereas the Tongan participants preferred the agent/water as causative with an average rating of 68.3% (corresponding to 31.7% wood [26.3; 36.9]). Given that the relation under consideration is physically symmetric, implying equal contribution of the two entities, both the Tongan and the German response patterns exhibit an asymmetry, albeit in diverging directions. This finding is largely consistent with previous results obtained using the forced-choice response format (Beller et al., 2009b; Beller and Bender, 2015).

Beyond that, the two linguistic variations probed within each language—word order and verb type—also affected causal assignments: Emphasizing the agent by word order/subject position resulted in a preference for the agent/water as causative (59.0%; corresponding to 41.0% wood [95% CI: 34.7; 47.3]), while emphasizing the patient/wood resulted in a balanced rating centered around the midpoint of the scale (53.0% wood [46.7; 59.2]). A similar preference for the agent/water was found for transitive verbs (58.3%; corresponding to 41.7% wood [35.4; 47.9]), while using an intransitive verb resulted in a balanced rating (52.3% wood [46.0; 58.6]). The impact of linguistic cues is thus not restricted to Tongan, as was observed previously (Beller et al., 2009b, reproduced in Figure 2A).

However, word order and verb type seem to play different roles in the two languages and to contribute differently to the overall effects, as indicated by a separate analysis of variance for each language: Word order played a significant role in German [*F*_(1, 89)_ = 6.71; *p* = 0.011; η_*p*_^2^ = 0.070], indicating a distinctive preference for the patient/wood as causative (69.6%; [61.6; 77.5]) if emphasized by word order/subject position, as compared to a balanced response (54.9% wood [46.7; 62.9]) if the agent/water was emphasized by word order/subject position. On the other hand, verb type did not make much of a difference [*F*_(1, 89)_ = 0.99; *p* = 0.322; η_*p*_^2^ = 0.011]. For Tongan, the pattern was reversed: Here, word order did not play a strong role [*F*_(1, 174)_ = 2.54; *p* = 0.113; η_*p*_^2^ = 0.014], whereas verb type made a significant difference [*F*_(1, 174)_ = 7.24; *p* = 0.008; η_*p*_^2^ = 0.040], indicating a stronger preference for the agent/water as causative (76.1%; corresponding to 23.9% wood [15.9; 32.0]) if phrased transitively (marked by the ergative case), as compared to the preference for the agent/water (60.5%; corresponding to 39.5% wood [31.4; 47.6]) if phrased intransitively (absolutive case).

The exploratory intransitive (and in Tongan partly ergative) phrasing (3) was assumed to split agency assignment, leaving parts of the agency with the wood (for floating) and assigning the remainder to the water (for enabling the wood to float). And in fact, in both languages, the causal assignment for phrasing (3) falls between the average levels reached for the intransitive phrasing (4) with patient (wood) in first/subject position and the transitive phrasing (1) with agent (water) in first/subject position.

Finally, the two transitive ergative phrasings in Tongan (1 and 2) elicited, as expected, a strong preference for the agent (the water) as causative (76.0%; corresponding to 24.0% wood) as compared to the intransitive, non-ergative phrasing (4), which elicited a rather balanced rating [44.9% wood; *t*_(130)_ = −2.934; *p* = 0.002; one-tailed].

#### Causal assignments in the breaking setting

The breaking setting differed from the floating setting mainly insofar as it involved a proper agent (a man) in an otherwise physical setting (a glass breaking) in three of the four linguistic variants. It aimed at testing whether the presence of such an agent affects the pattern in causal assignments found in the floating setting. The specific event was described either by using a transitive or an intransitive construction, three of these emphasizing the glass by word order and subject position—phrasings (5), (6), and (7)—and one emphasizing the man (8). Causal assignments were assessed with two different tasks per person, the first using a forced-choice format and the second a rating format. As can be seen in Figures 2C and D, we found a uniform preference for the human agent as causative for all agentive phrasings in both languages, and a difference only for the non-agentive phrasing (8), which elicited a rather balanced assessment in German and a strong focus on the unknown agent or entity.

##### Forced-choice data

In a preliminary step, we checked for the non-agentive variant (8) whether it made a difference how the response option that was provided as alternative to the glass was formulated: personal as “somebody unknown” or non-personal as “something unknown.” This was not the case. A log-linear analysis (Kennedy, 1992) with *response type* (personal vs. non-personal) and *language* (German vs. Tongan) as independent variables and the frequency of the two response options “glass” vs. “somebody/something else” as dependent variable indicated only a main effect of language (*G*^2^[1] = 22.22; *p* < 0.001), and no other effects (all *G*^2^[1] < 2.00; *p* > 0.156). We therefore regarded it as justified to aggregate the data across these two types of response options for the further analysis.

Similar to the floating setting, the event was described using transitive vs. intransitive constructions, but this time, we had three versions that emphasized the glass (by word order and subject position) and only one that emphasized the man. To test the four syntactic variants for differences, we therefore performed a log-linear analysis with only the two independent variables *syntactic variation* (phrasings 5, 6, 7, vs. 8) and *language* (German vs. Tongan), and the frequency of the two response options “glass” vs. “the man/somebody else/something else” as dependent variable. The results indicated a main effect of the syntactic variation (*G*^2^[3] = 22.74; *p* < 0.001), a main effect of language (*G*^2^[1] = 11.50; *p* < 0.001), and an interaction of the two factors (*G*^2^[3] = 10.70; *p* = 0.013).

Participants largely preferred the agent/the man and not the patient/the glass as causative albeit to a differing extent across the four syntactic variations: phrasing (5) 10.7% glass; phrasing (6) 11.7% glass; phrasing (7) 10.3% glass; phrasing (8) 35.4% glass (see Figure 2C). The two main effects primarily resulted from a difference for the non-agentive phrasing (8) across samples, as indicated by the significant interaction: While most Tongan participants still regarded an (unknown) agent/factor as causative in this condition (18.0% glass; 82.0% unknown factor), the majority of the German participants now attributed the breaking of the glass to the glass itself (65.7% glass; 34.3% unknown factor).

##### Rating data

Participants' causal assignments were coded by measuring their marks on the scale accurate to 0.5 mm, ranging from 0 cm (0% glass), when the mark was precisely on the endpoint labeled with “the man” or “somebody/something unknown,” to 10 cm (or 100% glass), when the mark was precisely on the endpoint labeled with “the glass.” Accordingly, values above 50% indicate a stronger causal role of the patient/the glass and values below 50% indicate a stronger causal role of the (possible) agent/the man or somebody/something unknown.

Again, we checked in a preliminary step for the non-agentive variant (8) whether responses depended on how the alternative response option to the glass was formulated: personal or non-personal. This was not the case. An analysis of variance with *response type* (personal vs. non-personal) and *language* (German vs. Tongan) as independent variables and the rating of glass-as-causative as dependent variable indicated only a main effect of language [*F*_(1, 95)_ = 17.80; *p* < 0.001; $\eta_{\text{p}}^{2}$ = 0.158], and no other effects [all *F*_(1, 95)_ < 1.0; *p* > 0.521; $\eta_{p}^{2}$ < 0.004]. We therefore regarded it as justified to aggregate the data across these two types of response options for the further analysis.

To test the four syntactic variants for differences, we performed an analysis of variance with two independent variables, *syntactic variation* (phrasings 5, 6, 7, vs. 8) and *language* (German vs. Tongan), and the rating of glass-as-causative as dependent variable. The results indicated a main effect of syntactic variation [*F*_(1, 263)_ = 4.46; *p* = 0.004; $\eta_{p}^{2}$ = 0.048] and an interaction with language [*F*_(1, 263)_ = 4.67; *p* = 0.003; η_*p*_^2^ = 0.051], while the main effect of language did not reach significance [*F*_(1, 263)_ = 3.14; *p* = 0.078; η_*p*_^2^ = 0.012].

Similarly to the forced-choice data, yet slightly less extremely, participants mostly preferred the agent (the man) and not the patient (the glass) as causative, albeit to a differing extent across the four syntactic variations: phrasing (5) 21.6% glass [95% CI: 13.1; 30.1]; phrasing (6) 25.4% glass [16.9; 34.0]; phrasing (7) 20.4% glass [11.9; 28.9]; phrasing (8) 37.1% glass [30.7; 43.6] (see Figure 2D). Bonferroni-corrected comparisons between the four syntactic variations did not indicate any difference (all *ps* > 0.167). Assignment of causality to the breaking glass was again highest for the non-agentive version (8), and this effect was again due to the German participants, as indicated by the significant interaction.

##### Summary

With one exception, German and Tongans alike assigned prime causality to the person involved and not to the object. Similarly, with one exception, none of the linguistic variations had any effect. The exception to both overall patterns is the non-agentive phrasing (8) which led a substantial proportion of our German participants to switch their causal assignment from the human agent to the patient, while it did not affect the response of our Tongan participants at all. This latter finding implies that ergativity had no effect in this case. The German pattern is thus consistent with findings reported by Fausey and Boroditsky (2010), in which a non-agentive phrasing also decreased causal assignment to the agent by English speakers.

At first glance, the German pattern is also consistent with our assumption put forward above that shifting the patient (i.e., the glass) to the subject position in phrasing (8), marked by the nominative case in German, may endow it with agent-like properties and hence be responsible for this switch in causal assignment. This interpretation is weakened, however, by the results of phrasing (7) with patient shift. The German version of phrasing (7) preserves the intransitive structure of (8) together with the linguistic marking of “the glass” as subject in the nominative case, but simply adds “the man” as the person affected by the breaking of the glass. This addition of (actually irrelevant) information suffices to switch the causal assignment “back to normal,” hence rendering the man as the cause (for related effects of additional yet irrelevant information on causal assignments, see also Beller and Bender, 2015). In other words, mentioning a possible agent, even if not in a linguistically prominent position, appears to shift agency assignment in German toward this candidate^2^.

#### Comparison of verb causality across languages and stimuli

Part 2 aimed at exploring language-specific effects of verb semantics on causal assignments, which would also allow us to assess possible interferences of verb semantics with the syntactic effects addressed in other parts of the survey. To this end, participants were asked to assign causality on a rating scale for minimal social scenarios based on different verbs. The first group of items consisted of 12 verbs that can be used to describe physical settings (see Table 2, 1–12). The second group consisted of four verbs referring to social transactions, which are highly valued in Tongan culture (Table 2, A to D). The goal of this task was exploratory in nature, and we assumed that the social verbs are more likely inclined toward an object focus in Tongan than in German.

**Figure 3:**
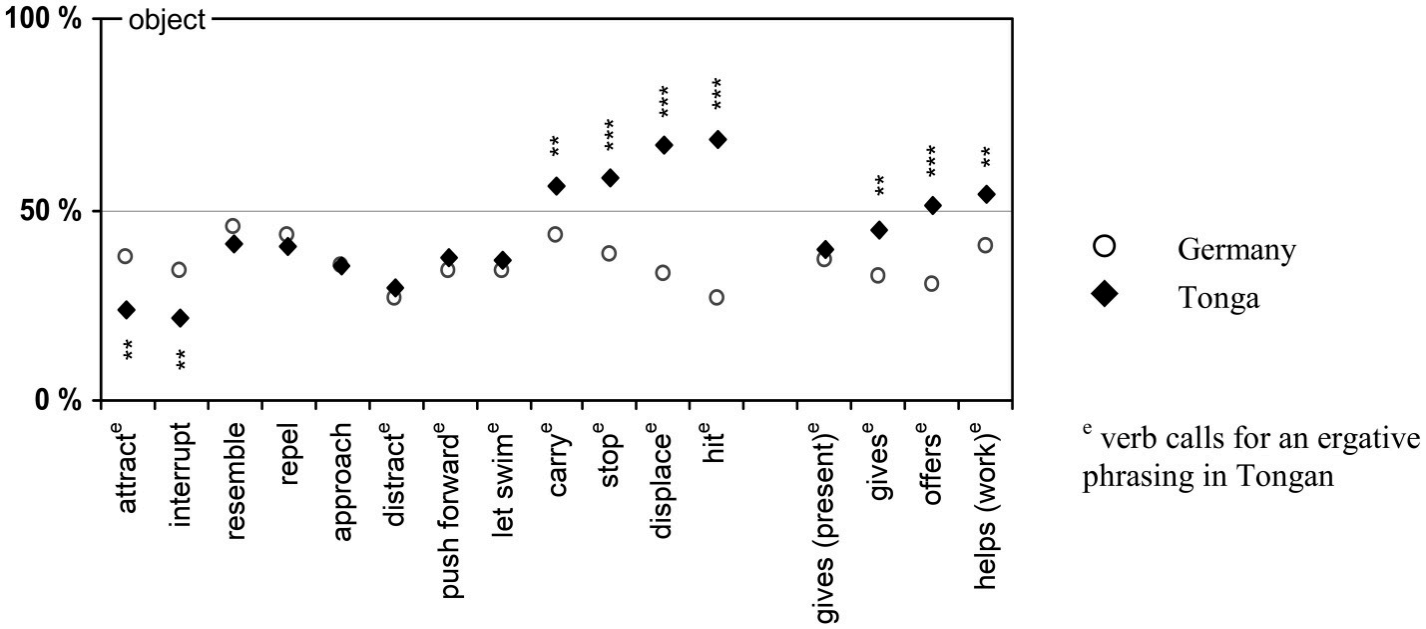
Assignment of causality in Part 2 on verb causality.

The causal assignments were coded by measuring participants' marks on the rating scale accurate to 0.5 mm ranging from 0 cm (0% object) when the mark was precisely on the endpoint labeled with the name of the person in the *subject* position, to 5 cm (or 100% object) when the mark was precisely on the endpoint labeled with the name of the person in the *object* position. Accordingly, values above 50% indicate a stronger causal role of the patient/object and values below 50% indicate a stronger causal role of the agent/subject. As indicated in Figure 3, participants' causal assignments varied with the verb, but differently across the two languages.

To test effects of implicit verb causality, an analysis of variance was performed with the independent variable *language* (German vs. Tongan) and a repeated measurement across the ratings of the 16 verbs. The results indicated main effects of the factors language [*F*_(1, 247)_ = 15.75; *p* < 0.001; η_*p*_^2^ = 0.060] and verb [*F*_(13.5, 3337.1; Greenhouse − Geisser corrected degrees of freedom)_ = 10.80; *p* < 0.001; η_*p*_^2^ = 0.042], and an interaction of the two factors [*F*_(13.5, 3337.1)_ = 11.68; *p* < 0.001; η_*p*_^2^ = 0.045].

In general, both German and Tongan participants revealed a subject focus, albeit in different proportions: It was stronger for the German participants who assigned less responsibility to the person in the object position (35.5% [95% CI: 32.0; 39.0]) than the Tongan participants (44.3% [41.7; 47.1]). In addition, there was variation across the 16 verbs with ratings for the object as causative ranging from 28.2% ([23.8; 32.6]) for “interrupt” to 49.8% ([45.1; 54.6]) for “displace,” but the causal assignments for the verbs interacted with language. In the German sample, the assignments for all verbs were significantly below 50%, thus indicating a subject focus [largest *t*_(92)_ = −2.014; *p* = 0.047; one-sample t-test; two-tailed]. In the Tongan sample, three verbs showed a significant object focus with assignments larger than 50% [“stop,” “displace,” and “hit”; smallest *t*_(176)_ = 2.446; *p* = 0.015], four verbs showed balanced assignments not significantly different from 50% [“carry,” “gives,” “offers,” and “helps (work)”; largest |*t*_(174)_| = 1.901; *p* = 0.059], whereas all other verbs showed a significant subject focus [largest *t*_(175)_ = −3.102; *p* = 0.002].

For six verbs of the first group, a subsequent *t*-test indicated cross-linguistic differences [smallest |*t*_(266)_| = 2.541; *p* = 0.012; two-tailed]. The verbs “attract” and “interrupt” showed a stronger subject focus in Tongan than in German, whereas the verbs “carry,” “stop,” “displace,” and “hit” showed an object focus in Tongan, but a subject focus in German. This finding is particularly noteworthy, as each of these latter four verbs would likely be considered an *action-agent verb* according to Au's (1986) terminology. This raises the question of whether the verbs entail different connotations across languages as part of their semantic meaning, or whether culture- and/or language-specific concepts additionally affect the interpretation of (otherwise similar) words. For the remaining six verbs of this group, no linguistic differences were found [largest |*t*_(260)_| = 1.273; *p* = 0.204; two-tailed].

Previous research in Tonga had suggested that giving and helping are considered as a response to what another person needs or requests, and may thus entail a stronger object focus in Tongan than in German. And indeed, the ratings for three out of the four respective verbs (“give,” “offer,” and “help”) differed significantly across languages. In each case, the German verb shows a subject focus, whereas its Tongan counterpart had a tendency toward the object [smallest |*t*_(267)_| = 2.532; *p* = 0.006; one-tailed]. The ratings for the fourth verb “gives [as a present]” did not differ [*t*_(265)_ = −0.640; *p* = 0.261; one-tailed]. Although the effects of cultural value are weaker than we expected them to be, these findings do provide good reasons to devote more attention to such effects in future research.

Finally, not all Tongan translations of transitive German verbs are transitive themselves. Roughly two thirds of the verbs scrutinized in Part 2 require the ergative for the subject (e.g., “hit”: *Ta'i 'e [S] 'a [O]*), while the remainder entail a prepositional construction (e.g., with *kia*, “to/toward,” as in *Fakaheleleu 'a [S] kia [O]*, “[S] interrupts [O]”). Do those verbs that require a subject in the ergative exhibit a stronger subject focus than those that do not require the ergative? While our list of verbs is neither comprehensive nor representative enough to justify broad generalizations, the results still reveal a pattern, but the trend is *contrary* to what we expected: Overall, the verbs requiring an ergative construction not only have a stronger focus on the object than their German counterparts, but also a stronger such tendency than the verbs that require a prepositional construction.

The case of “carry” is particular interesting in this regard, as it is the one verb that allows a comparison across Parts 1 and 2. While in Part 2, the abstract test of verb semantics suggests that “carry” evokes a subject focus in German and tends to evoke an object focus in Tongan, the assignments for “water carries wood” (phrasing [1] in Part 1) exhibited the opposite pattern: Here, Tongan participants were more strongly inclined to assign causality to the subject/water and German participants to the object/wood. In other words, the causality implicit in the verb “carry” has likely dampened a cross-linguistic difference in causal assignments that otherwise may have been even more pronounced. It is thus imperative that future work on causal scenarios, and especially so cross-linguistic research, takes implicit verb causality into consideration.

#### Possible limitations of the study

As mentioned above, the data reported here was part of a larger screening study, which may have two critical implications. First due to the exploratory purpose of the study, we did not scrutinize strong hypotheses, but were interested in probing the potential for cultural influences (including influences by linguistic properties) on causal cognition in the physical domain, where previous research has almost entirely neglected such a potential. Our findings are therefore preliminary and an indication of, rather than strong evidence for, such influences in the physical domain. Second, the fact that the tasks reported here were part of a larger study also implied limitations with regard to the number of items that could be tested and the number of permutations that were possible. This constrains the generalizations we can draw form our findings. For the sake of feasibility of the whole study, for instance, we dispensed with a second version of the floating setting with a forced-choice format, as we already had partial data on it, and we dispensed with a more complete permutation of the content variations. As a consequence, only tentative inferences can be drawn from comparing the different settings on floating and breaking and their response format (Figure 2).

Yet, while both, the shortness in strong hypotheses and the limited comparability across conditions, prevent us from drawing straightforward inferences, the data presented here still suggest that causal cognition in the physical domain is susceptible to cultural and linguistic influences, hence justifying more thorough and in-depth investigations in this direction. Such future research should then also investigate more thoroughly the manner in which these linguistic factors are affecting causal assignment (e.g., by casing, word order, or grammatical hierarchy).

In addition, one of the reviewers raised the question of whether our tasks may reveal more about language comprehension of our participants than about their cognitive processing of the scenario. In the classical study by Loftus and Palmer (1974), for example, participants' verbally reported memories of an event could be compared to the actually observed event as an objective reference, thus allowing for a strong test of how language may bias recall. In contrast, our own study integrated event information in the task to be conducted, couching this information in terms that already contain the linguistic cues under scrutiny. In other words, participants may have simply responded to the question by reflecting the presumptive meaning of phrasings like “carries” vs. “lets float” in their ratings (for an overview on presumptive meanings, see McCawley, 1978; Levinson, 2000). Yet, even if they did simply respond to the presumptive meaning conveyed by the linguistic cues, this would still be an interesting finding as it revealed that the underlying concept of, for instance, why wood floats on water is susceptible to such modification. Comparing across different variants of a task (for tasks of the same content) and across different content (for variants of the task) still allows us to disentangle effects of linguistic cues and of content at least to some extent. And indeed, the effects observed here did not simply reflect the linguistic cues, but additionally depended on the content of the scenario.

Another concern with the study arises from the differences in average age between the samples. For two reasons, we do not consider this critical. The first reason is that formal education does not prevent people from falling prey to the asymmetry bias (White, 2006, 2007). In the tasks we used here, the older German participants exhibited similar degrees of asymmetry as the younger Tongan participants although not always in the same direction. Second, the data on the floating setting presented in Figure 2 were actually collected with two different samples, the rating data (Figure 2B) in the study reported here and the forced-choice data (Figure 2A) in a previous study (Beller et al., 2009b). The German participants in that previous study were as young as the Tongan ones in the current study; still, their response pattern was similar to the (older) German participants in the current study and significantly different from the Tongan participants of the same age.

A final concern revolves around the translatability of the material and raises the question of whether, for instance, the verbs used really mean the same in the two languages under scrutiny. This concern is fueled by the findings from Part 2 on implicit verb causality, which indicated substantial differences in causal assignments even in the absence of context information. If, however, a verb invites causal assignment to the agent in one language, yet to the patient in another, the two may entail different connotations as part of their semantics, and hence may not be equivalent in meaning. This implication of our findings deserves to be taken seriously in future research in this field.

## General discussion

The prime objective of the study reported here was to explore whether and how language *per se* may affect causal cognition in the physical domain, and how differences between languages may come to bear on these effects. Three potentially relevant factors were targeted: (i) syntactic structure, (ii) the presence or absence of a full-fledged semantic agent, and (iii) the causality implicit in verb semantics. Despite the exploratory nature of the screening, the findings presented here still point toward intra- and cross-linguistic effects on causal assignments that are both interesting and important.

In the floating setting without agent, both speakers of German and of Tongan exhibit biases in their causal assignments, but in diverging directions, with German speakers favoring the patient and Tongan speakers favoring the agent, thus largely replicating a pattern found earlier (Beller et al., 2009a). These assignments are susceptible to syntactic cues such as transitive constructions and prior position in word order. In the breaking setting involving an animate agent, on the other hand, speakers of German and Tongan alike assign agency primarily to the agent, and almost irrespective of linguistic cueing—except for the non-agentive phrasing (8), for which speakers of German again shift toward the non-agent. And finally, while almost half of the verbs considered here do share implicit notions of causality across languages, thereby triggering similar causal assignments, the other half differ significantly, exhibiting a stronger object or patient focus on average in Tongan than in German, and more so for the “social” than for the “physical” verbs.

In the following, we discuss these main findings with respect to three issues: the domain-specificity of causal cognition, the ambiguous role of the ergative, and more general differences between languages.

### Physical vs. social settings: how dependent are effects on content domain?

A popular assumption, particularly among developmental psychologists, holds that causal cognition is domain-specific (e.g., Hirschfeld and Gelman, 1994; Spelke and Kinzler, 2007; and see Morris and Peng, 1994). However, the extent to which causal assignments in the physical domain differ from those in the social domain on principle grounds is still subject to debate. For instance, while attribution biases appear to occur both in social (Gilbert and Malone, 1995; Norenzayan and Nisbett, 2000) and physical scenarios (Peng and Knowles, 2003; White, 2006, 2007; Beller et al., 2009b; Bender and Beller, 2011), it has remained unclear whether these two are in fact equivalent (Malle, 1999; Sabini et al., 2001; White, 2006). The involvement of agents complicates matters even further: Whereas physical settings typically involve inanimate objects, the occurrence of a full-fledged animate agent is normally restricted to social or at least mixed settings. This has serious implications: First, with an agent, there is typically an inherent and strong asymmetry between participants in a state of affairs (e.g., someone hits someone, someone breaks something). How these thematic roles can be applied to symmetric relations that are at stake in most physical interactions has thus remained an unresolved question until recently (Mayrhofer and Waldmann, 2014). And second, if animate beings or even social actors are involved, they might attract more responsibility ascription *per se* than inanimate objects due to their greater causal effectiveness and self-reflexiveness (Leslie, 1996). This could also explain why a comparison of verb causality for interpersonal events and physical transfer events revealed significant differences (Majid et al., 2007).

In each of the two parts of our study, the two domains were compared at least indirectly. Although both the floating setting and the breaking setting of Part 1 on syntactic variations deal with physical situations and thus do not allow for strong conclusions across domains, the introduction of a proper agent in the breaking setting adds a different quality. And although some of the differences between the two tasks may be content-specific, at least the following aspects are noteworthy: First, striking cross-linguistic differences occurred across the board in the floating setting, but not in the breaking setting, where the two groups differed for one syntactic variation only. And second, while linguistic variations did have an effect in the floating setting, even if rather weak, this effect largely disappeared (again with one exception) in the breaking setting.

These differences can be explained in reference to the personal agent and in a related manner. The floating setting describes a symmetric physical relation, and although people tend not to perceive the symmetry (White, 2007)—with perception apparently being skewed by culture-specific concepts (Bender and Beller, 2011)—it may still trigger a sensation of ambiguity in at least some of the participants. In such a state, additional cues would be considered helpful to resolve the ambiguity and to come to a decision. The breaking setting, on the other hand, is causally more structured *a priori*, as it involves an animate agent. In this case, no ambiguity arises that would have to be resolved by linguistic cues; the social domain simply dominates the physical domain.

This may even be true for the exceptional case (8) for which the German participants assigned responsibility to the glass (rather than an unknown agent). As suggested by one of the reviewers, this specific sentence may have invoked notions related to a property of glasses, namely that they break easily, rather than notions related to a specific event. While such a property notion is more likely evoked by sentences that use the indefinite noun and a modified verb (as in “Glas bricht leicht” = *glass breaks easily*), the phrasing chosen here is still compatible with such a reading.

The findings of Part 2 on verb causality are more difficult to interpret in this respect. Our selection of verbs is somewhat skewed in comparison to the range that is typically explored in these kinds of studies because the prime goal of this part was to collect data on verb causality for verbs that can be used to describe physical relations. Furthermore, the tasks were implemented as minimal *social* scenarios, in line with the tradition in this field of research. Even if only *minimally* social, this social framing is not sufficiently abstract to prevent content effects (Majid et al., 2007), and it prevents inferences on how these verbs would have behaved in a purely physical context. Another consequence of the testing in minimal social scenarios was that some verbs shifted in meaning when transferred from the physical to the social domain. For instance, the German verb *anziehen* (“to attract”)—besides inviting the meaning of “to dress”—refers to different events depending on whether the entities involved are celestial bodies like earth and moon or are human beings, where the term gains a distinctively emotional aspect. In order to be able to address questions of domain-dependence in verb semantics, better controlled experiments are clearly needed in future research.

### Agent vs. patient focus: the ambiguous role of the ergative

The main assumption behind our interest in effects of syntactic cues was that differences in the relational structure of languages may affect causality assignment; more specifically, speakers of an ergative language may pay more attention to agents that are marked by the ergative. Previous work examining speakers of an ergative language (Duranti, 1994), linguistically untrained deaf children (Goldin-Meadow, 2003), and eye movements of adult English speakers (Griffin and Bock, 2000) indicated that a patient focus, as inherent in ergative languages, may be a default bias both in attention and language production (Goldin-Meadow, 2003, p. 517): Action or event and the entity directly affected by it attract most of the attention, while agent information is an optional add-on. If, by default, speakers of an ergative language are generally used to receiving information about the action and the entity affected, then introducing a (transitive) agent and marking him or her with the ergative case by way of exception should serve as a particularly potent tool for agency assignment. This is, in fact, what has been observed, for instance in socio-political discourse in Samoa (Duranti, 1994). The observation that, on average, our Tongan participants assigned causality largely—and more strongly so than the German participants—to the agent in each of the two settings (i.e., to the water in the floating setting, and to the man or something/somebody unknown in the breaking setting) would be in line with this hypothesis.

Further support was provided by a study on physical settings, where a change from an intransitive description (phrasing [4] “wood floats on water”) to a transitive phrasing with the water in the ergative (phrasing [2] “wood is carried by water”) shifted causal assignments among our Tongan participants more toward the water (Figure 2A; Beller et al., 2009b). This pattern could be replicated, by and large, in the current study (Figure 2B)—although not any longer exclusively for the Tongan speakers, but now also for the German speakers with their non-ergative language. Across the two languages, however, the same effect may also be accounted for by changes in word order, which prevents us from drawing strong conclusions on exactly which linguistic cue is responsible for the shift in assignment.

Moreover, the pattern described above could *not* be replicated in the breaking setting, where the obvious presence of an agent apparently eliminated the seductive effect of a (rather subtle) linguistic cue such as the ergative. Here, it seems as if the default focus on event and patient in ergative languages immunizes their speakers against the adding of information about the agent. Nominative-accusative languages like German, on the other hand, override the patient focus with their accusative structure and may thus sensitize their speakers to the presence or absence of agency information.

The pattern observed for implicit verb causality seems to suggest an interaction with the ergative, but in the *opposite* direction, with those verbs which afford an ergative phrasing being even more strongly object-focused on average than those which do not afford an ergative. However, the sample of items was non-representative and certainly too small to draw strong conclusions from the findings.

Given this mixed pattern of findings, it is difficult to decide whether the presence or absence of ergative case-marking in a given phrasing is actually strong enough a cue to increase or decrease the likelihood of assigning causal power to the agent. Currently, the data from the floating setting—which, with its symmetric configuration and the experimental variation of linguistic cues, can be considered the most informative task for this question—seems to support the former interpretation rather than the latter. Beyond these intra-linguistic cues, however, it seems still plausible that the relational structure of the language (e.g., whether agents of transitive constructions are singled out by specific case marking) may increase the salience of agency as one of their relevant properties.

### Across languages (and cultures): how diverse is causal cognition?

Teasing apart the influences of culture and language on cognition is by no means a trivial undertaking. Not only is language an essential and integral part of culture, which bedevils any attempt to conceptually distinguish the two; it is also challenging to separate them methodologically (Beller et al., 2015; Iliev and Ojalehto, 2015). With the tasks used here, for instance, it is almost impossible to assess whether the stronger object focus on average for the socially salient transactions in Tongan is caused by the cultural value linked to these transactions or whether it has become part of the semantics of the verb. The situation is somewhat clearer with regard to the causal assignments for the physical settings, were the difference between samples (cultural groups) is greater than the difference between conditions (linguistic cues). This does not, however, resolve the question of whether the differences between samples are based on linguistic encoding or cultural entrenching in the first place. In other words, while the relational structure of one's language may affect how people perceive or assign agency, we cannot currently rule out that their respective tendencies are also, or perhaps exclusively so, shaped by culture-specific concepts linked to the setting under scrutiny.

This conceptual question aside, at least some general conclusions with regard to diversity and universality in causal cognition can still be drawn (cf. Beller et al., 2014). Across the board, we found both shared and distinct patterns. The two groups *resemble* each other in that they exhibit biases when assigning causality in the symmetric floating setting, in that (and in how) they respond to linguistic cues, and in most assignments of causality in the breaking setting. They *differ* in the direction of some of these biases, for instance in the floating setting (for other scenarios, see also Bender and Beller, 2011), in how they interpret non-agentive phrasings in the breaking setting, and in how they assign agency in some of the minimal social scenarios. While the similarities seem to support assumptions on general reasoning tendencies (e.g., White, 2006, 2007), the subtle yet pervasive differences between the two groups also point toward a susceptibility of these tendencies to external influences. Given the linguistic variations in Part 1 of our study, for instance, it appears likely that culture-specific schemas of agency and causation shift the focus of attention either more toward the agent or more toward the patient.

Currently, no available theoretical approach is able to account for this. The *proto-agency theory* (Dowty, 1991; Mayrhofer and Waldmann, 2014), for instance, identifies a set of properties on which agency assignment may be based. This might account for the similar patterns in the breaking setting, where “the man” garners three of these properties (i.e., independent existence, sentience/perception, and causation of a change of state), while the scenario remains silent on the other two (volition and movement). It may even be compatible with the difference for the non-agentive scenario (phrasing [8] “the glass breaks”), namely when assuming that participants differ in whether or not they imagine a person as part of the scenario. However, the *proto-agency theory* cannot (yet) explain the cultural differences in the floating setting, where the causal assignments of German and Tongan speakers co-vary with the manipulations in linguistic cues, but generally differ in the entity on which they focus as mainly causative. For a better understanding of such cultural influences, we thus not only require more empirical data, but also advancement in theoretical models (Beller and Bender, 2017).

## Conclusion

Despite the relevance of causal cognition as a core topic for the cognitive sciences, previous research has paid only incidental attention to culture as a possibly constitutive factor (Bender et al., 2017)—a desideratum that is only slowly being addressed (e.g., by the contributions to Beller et al., 2014). In the two parts of the screening study reported here, we intended to explore the potential for cultural and linguistic influences by addressing two related questions: Are assignments of causal role domain-specific (by contrasting settings that do vs. do not involve a human agent), and are they affected by emphasis on the conceptual agent (by varying linguistic cues related to agency)? Our findings suggest that such linguistic cues do affect how people represent and explain causal facts and events, but that language-specific properties may also contribute to differences in people's responses. Assumptions about the mechanisms generating these differences are necessarily tentative, as the study was exploratory and its design did not warrant conclusive inferences. One of the questions still open for future research is whether what we found primarily reflects what people perceive vs. what they express. The most important contribution of the current study to the field is therefore that it has demonstrated the susceptibility of causal cognition to cultural and linguistic influences, even in the physical domain, and that it has identified some of the factors worth investigating more thoroughly.

## Author contributions

All authors listed have made a substantial, direct and intellectual contribution to the work, and approved it for publication.

### Conflict of interest statement

The authors declare that the research was conducted in the absence of any commercial or financial relationships that could be construed as a potential conflict of interest.

## Footnotes

^1^Please note that part of the data in Panel (B) overlaps with Figure 2B in Bender and Beller (2011).

^2^Actually, the non-agentive phrasing (8) had also mentioned a possible agent, albeit only indirectly through the two response options given: Half of the participants were offered a choice between “the glass” and “something unknown” (two non-personal options), and the other half were offered a choice between “the glass” and “somebody unknown,” with the latter providing a *personal* option. However, the different response options did not affect participants' causal assignments (as indicated by the preliminary analyses), and the option “somebody unknown” did not prime our German participants toward assuming this person to be the prime cause to the same extent as did the other phrasings.
